# Nonradiation-to-endoscopist ERCP is non-inferior to standard ERCP

**DOI:** 10.1007/s00464-021-08822-2

**Published:** 2021-10-26

**Authors:** Wei Zeng, Jie Hu, Yanglin Pan, Mingqing Zhang, Li Xu

**Affiliations:** 1 Department of Gastroenterology, Xiang’an Hospital of Xiamen University, School of Medicine, Xiamen University, 2000 Xiang’an East Road, Xiamen, 361102 Fujian China; 2 Department of Medical Engineering, The 305 Hospital of PLA, Beijing, China; 3 State Key Laboratory of Cancer Biology and National Clinical Research Center for Digestive Diseases, Xijing Hospital of Digestive Diseases, Air Force Medical University, Xi’an, 710032 Shaanxi China; 4 Department of Gastroenterology, 909 Hospital of PLA, Affiliated Southeast Hospital of Xiamen University, 269 zhanghua middle road, Zhangzhou, 363000 Fujian China

**Keywords:** Nonradiation-to-endoscopist (NRE) ERCP, Standard ERCP, Retrospective study, Safety

## Abstract

**Background:**

Radiation exposure is inherently involved in endoscopic retrograde cholangiopancreatography (ERCP), which could cause radiation-induced injury to endoscopists with long-term exposure. Nonradiation ERCP has been applied to pregnant patients. Conceivably, the same techniques could be used to benefit endoscopists. This study was designed to evaluate the effectiveness and safety of nonradiation-to-endoscopist (NRE) ERCP, compared with standard ERCP.

**Methods:**

A retrospective, single-center study was conducted from August 2010 to December 2015. Patients aged 18–90 years and with choledocholithiasis (< 15 mm) or distal biliary stricture were eligible. Pre-ERCP evaluation with magnetic resonance cholangiopancreatography was mandatory. To overcome selection bias, we performed 1:2 match using propensity score matching (PSM) between NRE and standard groups. The primary endpoint was overall ERCP success rate. Secondary endpoints were cannulation success rate, stone clearance rate, complication rate, and duration of hospitalization.

**Results:**

A total of 329 patients met inclusion criteria. After PSM, 73 patients were included in the NRE group and 146 in the standard group. The ERCP overall success rate for NRE and standard groups was equivalent (94.5% vs. 93.2%, *P* = 0.70). There was no difference in cannulation success rates between the two groups (95.6% vs. 97.8%, *P* = 0.39). A total of 88.3% of patients in the NRE group and 93.9% of patients in the standard group had stones cleared at initial ERCP (*P* = 0.57). No difference in overall stone clearance rate between the two groups (95.0% vs. 93.9%, *P* = 0.77) was found after second ERCP. The complication rate (1.4% vs. 1.4%, *P* = 1.00) and hospital duration (8.3 ± 5.1 vs. 10.2 ± 8.8 days, *P* = 0.07) were not different between the two groups.

**Conclusion:**

Although technically demanding, NRE-ERCP is both safe and feasible in selected patients compared with standard ERCP.

Endoscopic retrograde cholangiopancreatography (ERCP) is currently an established therapeutic technique for the treatment of lesions in the pancreatic or biliary duct and relies heavily on the use of fluoroscopy. ERCP endoscopists are therefore at risk for exposure to ionizing radiation over many years. While there is a general lack of data and of long-term follow-up data in particular, there is increasing awareness that accumulating doses of ionizing radiation from modern radiologic procedures may have harmful impact on health [1–3].

ALARA is a term representing the use of radiation dose “as low as reasonably achievable [4, 5].” Previous studies have focused on reducing radiation exposure to patients during ERCP [6–9]. Also, to protect endoscopists from radiation exposure, conventional methods, such as optimizing fluoroscopy unit variables (e.g., pulse rate, collimation, magnification), increasing the physical distance from the primary radiation source, using appropriate shielding, like Radiation-Attenuating Drape [10] and use of exposure detection programs [5, 10], have been described. Binmoeller et al. [11] reported the first case of nonradiation ERCP in pregnant women. Nevertheless, the reports of using nonradiation ERCP are limited and mainly used to solve uncomplicated choledocholithiasis [12, 13]. With the development of endoscopic technology, other nonradiation ERCP has been reported recently, such as endoscopic ultrasound (EUS)-based ERCP and ultrasound-based ERCP [14, 15]. However, these techniques not only require endoscopists to master both ultrasound and endoscopy technologies, they inferior with standard ERCP, especially in complex cases [16], which have limited their wide-spread application.

We presented our experience of performing nonradiation-to-endoscopist (NRE) ERCP. Contrary to standard ERCP that routinely use fluoroscopy for real-time observation, radiation was not only applied at limited specific situations but also used while endoscopists left the operating room to avoid exposure. The non-use of radiation to endoscopists during ERCP stands out as our major modification to the standard approach.

## Materials and methods

### Study design

This is a retrospective, case–control, single-center study conducted at the No.174 Hospital of the People’s Liberation Army, from August 2010 to December 2015. To overcome selection bias, a propensity score matching (PSM) study protocol was used between NRE and standard ERCP groups. Informed consent was approved by the institutional review committee of the No.174 Hospital of the People’s Liberation Army. The study was registered at Clinical Trials.gov (NCT02697149).

### Patients

Patients aged 18–90 years and who were undergoing ERCP were enrolled. Exclusion criteria included: (1) common bile duct stone (CBDS) > 15 mm; (2) complicated stricture defined as Bismuth III and IV and multiple strictures; (3) distal biliary stricture combined with CBDS; (4) patients with altered anatomy or uncertain biliary stricture. Endpoints comparison was done between patients in the NRE group with those in the standard group.

MRCP was mandatory for all patients enrolled. MRCP imaging facilitated the subsequent NRE-ERCP as a valuable anatomic guide. Informed consents for MRCP and ERCP were obtained after detailed counseling. In all cases, serum chemistry tests, including liver function (AST, ALT, and bilirubin) and amylase, and transabdominal ultrasound were conducted. Patients with suspected malignant biliary lesions received EUS-FNA or percutaneous biopsy, guided by ultrasound, before ERCP.

### Nonradiation-to-endoscopist ERCP procedures

All ERCP procedures were performed using a side-viewing duodenoscopy (JF-260; Olympus Co Ltd, Tokyo, Japan). An electrosurgical unit (ERBE IC-200) with a blended current and a power setting of 40 W was used. All ERCPs were performed by one endoscopist (ZW). Before start of NRE-ERCP study, Dr. Zeng has been engaged in standard ERCP operations for 7 years (2008–2015) and has completed more than 2000 cases in total. Therefore, he is skillful in the field of ERCP. Amylase levels were checked in all patients within 24 h after ERCP.

All devices used for NRE-ERCP and standard ERCP groups were same. The specific cannulation device, papillotomy knife, balloons, and guidewires used were at the discretion of the endoscopist and could be changed interprocedurally. Commonly used devices were Ultratome XL Triple Lumen Sphincterotome (Boston scientific, Trapezoid), guidewire (VisiGlide, Olympus), marked balloon (5 cm with red and 10 cm with green tape), and distance-marked wire-guided retrieval basket (Boston scientific, Trapezoid).

ERCP was performed with the patient placed in a left lateral to prone position. Moderate conscious sedation was achieved and maintained with intravenous propofol. Biliary cannulation was attempted using a triple lumen sphincterotome with a 0.035-inch super guidewire. Once deep cannulation was achieved, the guidewire was withdrawn and successful cannulation of bile duct was confirmed by visible bile in the catheter upon aspiration. The length of sphincterotomy was determined based on the diameter of the bile duct and the size of stones depicted at MRCP.

The strategies and methods after cannulation are summarized. For common bile duct stones less than 10 mm, stones were removed with a basket after Endoscopic sphincterotomy (EST); for common bile duct stones larger than 10 mm, balloon dilation and balloon retrieval were performed following EST. Uncovered metal stents were placed for malignant stenosis, while fully covered metal stents or plastic stents were used for benign stenosis. In facing of difficulty to pass the stricture, the distance between the stenosis segment and the duodenal papilla was first determined with MRCP and then wires were manipulated with the goal of advancing the wire freely and without resistance at various angles until deep access was achieved. ERCP catheters were then advanced over the wire and into the duct. Successful deep cannulation was judged by visible bile in the catheter with aspiration.

For NRE-ERCP, when fluoroscopy was used, it was only used under the following situations, all when endoscopist and assistants were out of the operation room: (1) if resistance was met when inserting guidewire; (2) after contrast is injected; (3) before all-in-one plastic stent is released. During the procedure, the endoscopist leaves the operating room to avoid exposure. An extension tube was used to connect the sphincterotome and syringe, and a micro pump was used for contrast agent injection. The injection was stopped once proper cholangiography was achieved.

### Data collection

Statistical data and clinical characteristics of all patients were collected. Patient-related data included gallbladder condition (with cholecystolithiasis, without cholecystolithiasis, resected versus not), intrahepatic bile duct stones, previous surgical history, comorbidities, clinical diagnosis, blood examination pre-ERCP, and papilla (normal, juxtapapillary diverticulum, inside the diverticulum). Procedure-related data included intubation techniques, stone extraction techniques (cholangiography, sphincterotomy (EST), stone basket, balloon extraction), dilation techniques (endoscopic papillary balloon dilatation (EPBD), endoscopic probes dilation), drainage techniques (endoscopic nasobiliary drainage (ENBD), stent placement), and other techniques (biliary biopsy, papilla biopsy). Other information, such as the maximum diameter of CBD, the number of stones, serum amylase within 24 h after ERCP, days of hospitalization after ERCP, and complications of ERCP, were all collected. All above data were assessed by one investigator (HJ).

### Statistical analysis

To overcome possible selection bias and potential confounders between NRE and standard groups, we performed one to two matching using PSM on Empower (R) software. This method tries to construct an RCT-like situation where observed outcomes can be compared between intervention groups. When performing PSM, six variables were included, such as gender, age, prior EST or not, gallbladder (with cholecystolithiasis, without cholecystolithiasis, having been resected), total bilirubin (TBIL), and diseases (CBDS ≤ 10 mm, CBDS > 10 mm, hilar stricture, non-hilar stricture, and others).

Categorical variables were analyzed using *χ*^2^ tests or Fisher’s exact test, as appropriate. Analyses were performed with SPSS software V.19.0 for Mac. *P* values of ≤ 0.05 were considered significant.

## Results

### Patient characteristics

**Fig. 1:**
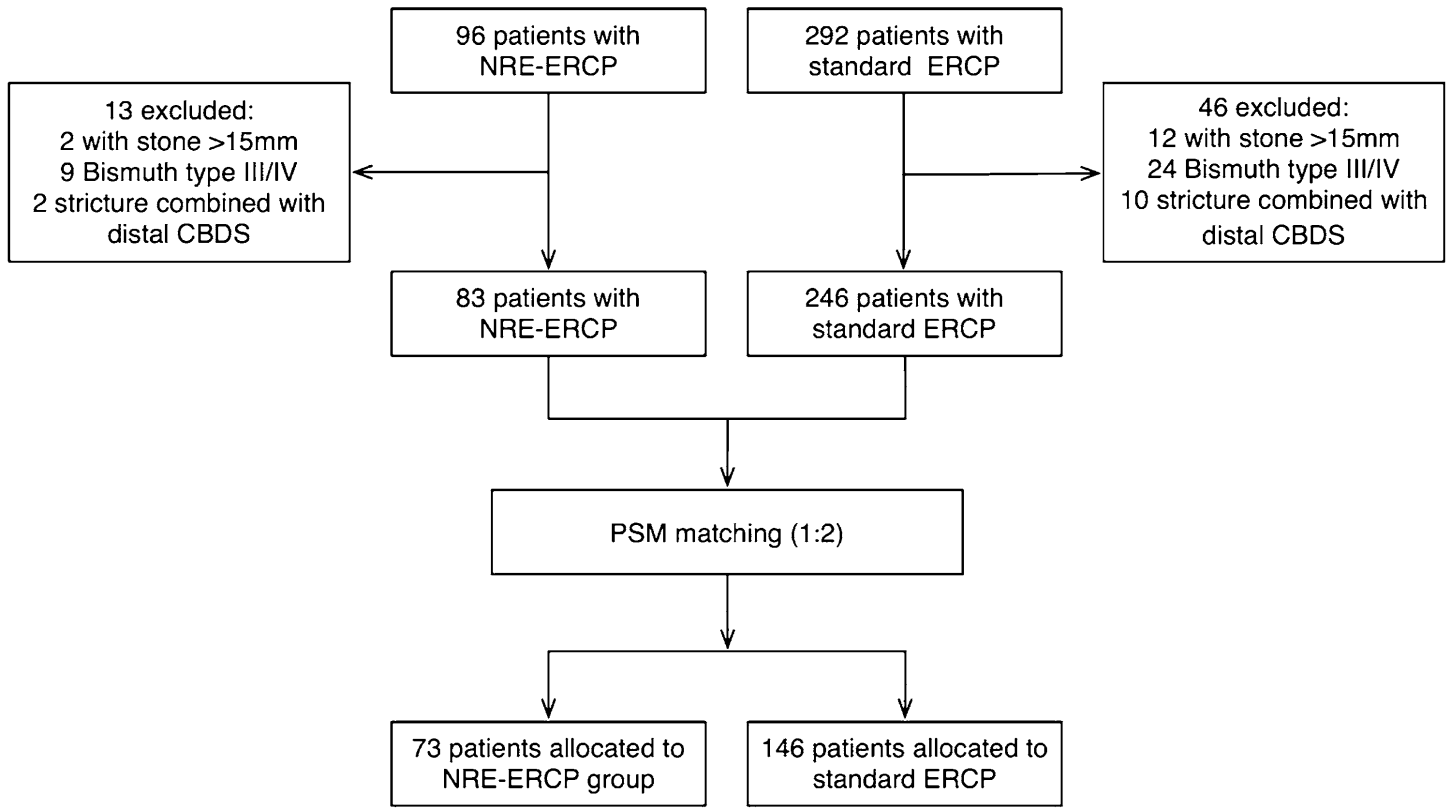
Flow chart of the study delineated the recruitment process. From August 2010 to December 2015, a total of 388 patients underwent ERCP in our endoscopy center, with 96 patients undergoing NRE-ERCP and 292 patients undergoing standard ERCP. 13 patients from the NRE group and 46 patients from the control group were excluded based on exclusion criteria. A total of 329 patients (83 in NRE and 246 in standard group) who were eligible for PSM. After PSM analysis, 73 patients in the NRE group were matched 1:2 to 146 patients in the standard group

From August 2010 to December 2015, a total of 388 patients underwent ERCP in our endoscopy center, with 96 patients undergoing NRE-ERCP and 292 patients undergoing standard ERCP. Thirteen patients from the NRE group and forty-six patients from the control group were excluded based on exclusion criteria. A total of 329 patients (83 in NRE and 246 in standard group) who were eligible for PSM. After PSM analysis, 73 patients in the NRE group were matched 1:2 to 146 patients in the standard group. Figure 1 delineates the recruitment process. All baseline characteristics were well balanced between the two groups (Table 1).

**Table 1:** Patient baseline characteristics in the NRE and standard ERCP groups

	NRE (*n* = 73)	Standard (*n* = 146)	*P* value
Age (y), mean (SD)	57.8 (14.7)	58.5 (14.6)	0.74
BMI (value),mean (SD)	20.96 (3.26)	21.86 (2.55)	0.29
Gender (male/female)	38/35	73/73	0.77
Prior ERCP	5 (6.8%)	10 (6.8%)	1.00
Prior EST	5 (6.8%)	7 (4.8%)	0.53
Indications for ERCP			
CBDS ≤ 10 mm	53 (72.6%)	100 (68.5%)	0.53
CBDS > 10 mm	7 (9.6%)	15 (10.3%)	0.87
Hilar stricture (Bismuth I/II)	5 (6.8%)	9 (6.2%)	0.85
Non-hilar stricture	7 (9.6%)	18 (12.3%)	0.55
Others	1 (1.4%)	4 (2.7%)	0.53
Papilla position			
Normal	60 (82.2%)	123 (84.2%)	0.70
Juxtapapillary diverticulum	12 (16.4%)	20 (13.7%)	0.59
Intra-diverticulum	1 (1.4%)	3 (2.1%)	0.72

*NRE* nonradiation-to-endoscopist; *SD* standard deviation; *ERCP* endoscopic retrograde cholangiopancreatography; *EST* endoscopic sphincterotomy; *CBDS* common bile duct stone

For the indications of ERCP, in the NRE group, 60 (82.2%) patients were diagnosed as common bile duct stone (CBDS), 12 (16.4%) were biliary stricture, and 1 (1.4%) was pancreatic cancer. In the standard group, 115 were CBDS (78.8%), 27 were biliary stricture (18.5%), and 4 were others (2.7%), 3 with acute pancreatitis and 1 with pancreatic pseudocyst.

### Endpoints

The primary endpoint was overall ERCP success rate. Secondary endpoints included cannulation success rate, stone clearance rate in the initial ERCP, overall stone clearance rate, complications after ERCP, and the duration of hospitalization.

The success and safety of an ERCP are heavily dependent on cannulation of the desired duct. There was no significant difference in the overall ERCP success rate (69/73, 94.5% vs. 136/146, 93.2%, *P* = 0.70) in NRE and standard groups, respectively (Table 2). Also, no significant difference of cannulation success rate between the two groups was identified (65/68, 95.6% vs. 133/136, 97.8%, *P* = 0.39). Although 12 and 27 patients were diagnosed stricture in NRE and standard groups, respectively, the successful cannulation rate through stricture was similar (11/12, 91.7% vs. 27/27, 100.0%, *P* = 0.24).

**Table 2:** Comparison of stone retrieval and clearance rates

	NRE (*n* = 73)	Standard (*n* = 146)	*P* value
Overall success rate of ERCP	69/73 (94.5%)	136/146 (93.2%)	0.70
Cannulation success rate	65/68 (95.6%)	133/136 (97.8%)	0.39
Success rate through stricture	11/12 (91.7%)	27/27 (100.0%)	0.24
Stone clearance at the initial ERCP	53/60 (88.3%)	98/115 (93.9%)	0.57
Stone clearance after the second ERCP	57/60 (95.0%)	108/115 (93.9%)	0.77
Clearance of small stone (≤ 10 mm)	51/53 (96.2%)	96/100 (96.0%)	0.95
Clearance of large stone (11 mm–15 mm)	6/7 (85.7%)	12/15 (80.0%)	0.75

*NRE* nonradiation-to-endoscopist; *ERCP* endoscopic retrograde cholangiopancreatography

With respect to stone clearance, a total of 53 of 60 (88.3%) of patients in the NRE group and 98 of 115 (93.9%) of patients in the standard group had stones cleared at initial ERCP (*P* = 0.57). After second ERCP, the total rates of stone clearance were 95.0% and 93.9% of patients, respectively (*P* = 0.77). Comparing the clearance rate in relation to the size of stones, similar clearance rates were observed for both small stones (≤ 10 mm) (51/53, 96.2% vs. 96/100, 96.0%, *P* = 0.95) and large stones (11 mm–15 mm) (6/7, 85.7% vs. 12/15, 80.0%, *P* = 0.75), respectively.

For all procedures, no difference was observed in naive papilla between NRE and standard groups (68/73, 93.2% vs. 136/146, 93.2%, *P* = 1.00). No difference of CBD size was observed between the two groups (11.5 ± 3.6 vs. 12.5 ± 4.9, *P* = 0.12) (Table 3). For stone extraction methods, performing EST after balloon dilation in the NRE group was more frequent than in the standard group (51/60, 85.0% vs. 74/115, 64.3%, *P* < 0.01), while the usage of stone basket in the NRE group was less frequent than that in the standard group (36/60, 60.0% vs. 87/115, 75.7%, *P* = 0.03). There were no differences in use of extraction balloon (51/60, 85.0% vs. 102/115, 88.7%, *P* = 0.49) and mechanical lithotripsy (ML) (0/60, 0.0% vs. 2/115, 1.7%, *P* = 0.53) between the two groups. Stent placements was done in 58.3% patients in the NRE group and 44.4% patients in the standard group had (*P* = 0.43).

**Table 3:** Comparison of procedural details between the two groups

	NRE (*n* = 73)	Standard (*n* = 146)	*P* value
Naive papilla	68/73 (93.2%)	136/146 (93.2%)	1.00
Stone number			
1	23/60 (38.3%)	58/115 (50.4%)	0.13
2	15/60 (25.0%)	16/115 (13.9%)	0.07
≥ 3	18/60 (30.0%)	29/115 (25.2%)	0.50
Sand like	4/60 (6.7%)	12/115 (10.4%)	0.42
Stone size (mm), median (range)	6.7 (2.7)	6.8 (3.4)	0.89
≤ 10 mm	53/60 (88.3%)	100/115 (87.0%)	0.79
10 mm–15 mm	7/60 (11.7%)	15/115 (13.0%)	0.79
CBD size (mm), median (range)	11.5 (3.6)	12.5 (4.9)	0.12
Methods of stone extraction			
EST after balloon dilation	51/60 (85.0%)	74/115 (64.3%)	< 0.01
Dormia basket	36/60 (60.0%)	87/115 (75.7%)	0.03
Extraction balloon	51/60 (85.0%)	102/115 (88.7%)	0.49
Mechanical lithotripsy	0/60 (0.0%)	2/115 (1.7%)	0.53
Procedure time (min), mean (SD)	50.26 (12.48)	40.49 (9.7)	0.83
Radiation exposure (sec), mean (SD)	206.92 (58.74)	266.31 (84.18)	0.042

*NRE* nonradiation-to-endoscopist; *EST* endoscopic sphincterotomy; *ERCP* endoscopic retrograde cholangiopancreatography; *SD* standard deviation

As the total procedure time included the time that the endoscopist went out and back the operation room when using fluoroscopy and sometimes increased attempts for certain procedure, patients in NRE group stayed in the operation room for longer time (average 9–10 min) compared with that in standard ERCP group. However, as the endoscopist did not perform the procedure under fluoroscopy, the radiation exposure time was significantly shortened in NRE group (206.92 ± 58.74 vs. 266.31 ± 84.18, *P* = 0.042) (Table 3).

Regarding complications after ERCP, there was no difference between the two groups (1/73, 1.4% vs. 2/146, 1.4%, *P* = 1.00) (Table 4). Specifically, in the NRE group, one patient was observed post-ERCP to have pancreatitis, while in the standard group, one patient had suspected perforation and one had cholangitis. No deaths occurred in either group. Moreover, there was no statistical difference in duration of hospitalization between NRE and standard ERCP groups (8.3 ± 5.1 vs. 10.2 ± 8.8, *P* = 0.07).

**Table 4:** Comparison of complications and mortalities between the two groups

	NRE (*n* = 73)	Standard (*n* = 146)	*P* value
Duration of hospitalization for CBDS patients (day), mean (SD)	8.3 (5.1)	10.2 (8.8)	0.07
Complication	1 (1.4%)	2 (1.4%)	1.00
Post-ERCP pancreatitis	1 (1.4%)	0 (0.0%)	0.27
Bleeding	0	0	NA
Perforation	0 (0.0%)	1 (1.0%)	0.80
Cholangitis	0 (0.0%)	1 (1.0%)	0.80
Mortality	0	0	NA

*NRE* nonradiation-to-endoscopist; *ERCP* endoscopic retrograde cholangiopancreatography; *CBDS* common bile duct stones; *SD* standard deviation

## Discussion

ERCP inherently involves radiation exposure, which has a recognized risk to both patients and medical personnel [17–19]. Although nonradiation ERCP was reported in patients in previous reports for uncomplicated choledocholithiasis, how to minimize the risk from cumulative effects of radiation to ERCP endoscopists remains unresolved in face of complicated cases. It remains unclear whether ERCP is also effective and safe when endoscopists were not exposed to radiation. In the current study, we reported and summarized our experience with nonradiation-to-endoscopist ERCP (NRE-ERCP) and with standard ERCP, allowing for a within institution comparison. To the best of our knowledge, it was for the first time that the idea of NRE-ERCP is proposed. We demonstrated that NRE-ERCP modality is both feasible and safe in experienced hands.

A guideline published by ESGE showed that the mean dosage to skin during ERCP ranged between 55 and 347 mGy. As radiation had an accumulative effect to health injury, it has been reported that once the total amount of accumulating doze over 100 mSv reached, it may decrease WBC, while dosages over 1000 mSv lead to vomiting and alopecia in a short period. Thus, avoiding radiation during ERCP performance is important to endoscopists and their assistants. Different from nonradiation ERCP that completely abandoned the use of fluoroscopy in the whole procedure, NRE-ERCP focused on the protection of endoscopists and assistants, as they are routinely having a high chance of being exposed to radiation.

In our study, ERCP, sphincterotomy, and common bile duct clearance were successful in all patients. MRCP was performed in all patients to confirm the presence of common bile duct stones and specify their number and location. It also helped to delineate the biliary anatomy and facilitate the upcoming ERCP. No post-ERCP major complications were noted in either group.

In our study, less exposure time was achieved by NRE-ERCP, and it led more total operation time as it took time for operator to go out and back the operation room and sometimes increased attempts for certain procedure. However, longer operation time did not lead to any extra complications for the patients as shown in Table 4.

The strengths and advantages of our study are as follows. First, to reduce selection bias, we adopted PSM in our data analysis method, instead of routine case–control study. Second, our results demonstrated that NRE-ERCP is feasible and necessary for a certain group of endoscopists and assistants, such as those who are in pregnant, those planning to have a child, or in lactating stage. Third, by avoiding the exposure to radiation, this procedure would reduce the accumulative injury effects of radiation to the health of endoscopists and assistants. Further, as we only took fluoroscopic images when needed, i.e., in contrast to its use in real-time fashion with standard ERCP, the total radiation exposure to patients is also likely reduced. Our findings support the notion that an NRE-ERCP approach will reduce radiation exposure to patients. They support the conduct of a future prospective study to measure the radiation exposure time and dosage to patients. Finally, unlike ultrasound-based ERCP, NRE-ERCP can be carried out directly without the need for additional equipment and new technology.

The present study has several limitations that warrant consideration. First, it is inherently limited by its nonrandomized, retrospective nature. Second, the performance of ERCP without fluoroscopy is technically demanding and a certain level of experience is required. Cannulation may be difficult, and confirmation of selective bile duct access can be challenging without the use of radiographic methods. Owing to the lack of real-time fluoroscopic monitoring, the range of treatable diseases is more limited than standard ERCP. It may not be feasible in cases of multiple stones (≥ 3), large stones (≥ 10 mm), and complicated stricture (Bismuth III and IV, multiple stricture). Third, in the procedure of NRE-ERCP, the success of cannulation was confirmed with the technique of bile duct aspiration. Mistakes might be made in judging the color of aspirated liquid in case of severe CBD obstruction.

In conclusion, from our retrospective study, we demonstrated that NRE-ERCP is a feasible and safe alternative technique compared with standard ERCP. It is designed to avoid radiation exposure to endoscopists and their assistances and patients when performing ERCP. Among selected patients, NRE-ERCP achieved similar overall success rate of cannulation and stone clearance, as that with standard ERCP. No additional complications or prolonged hospitalization duration were associated with NRE-ERCP compared to standard ERCP. For patients with choledocholithiasis (< 15 mm) and hilar bile duct stricture (stricture defined as Bismuth I and II), this technique has the potential to reduce radiation exposure to endoscopists, other medical staff, and patients. Nevertheless, although ERCP was safely completed without the use of fluoroscopy at our center, as the operator has extensive experience in the field of ERCP, patient safety cannot be assured when performing ERCP without the benefits of fluoroscopy at all ERCP centers. The current study warrants the conduct of future studies to specifically examine this. Moreover, studies from other centers are warranted to determine whether NRE-ERCP could be widely adopted.
