# Correction to: Nonradiation-to-endoscopist ERCP is non-inferior to standard ERCP

**DOI:** 10.1007/s00464-021-08869-1

**Published:** 2021-11-16

**Authors:** Wei Zeng, Jie Hu, Yanglin Pan, Mingqing Zhang, Li Xu

**Affiliations:** 1 Department of Gastroenterology, Xiang’an Hospital of Xiamen University, 2000 Xiang’an West Road, Xiamen, Fujian People’s Republic of China; 2 Department of Medical Engineering, The 305 Hospital of PLA, Beijing, China; 3 State Key Laboratory of Cancer Biology and National Clinical Research Center for Digestive Diseases, Xijing Hospital of Digestive Diseases, Air Force Medical University, Xi’an, 710032 China; 4 Department of Gastroenterology, 175 Hospital of PLA, Affiliated Southeast Hospital of Xiamen University, Zhangzhou, 363000 Fujian Province China

## Correction to: Surgical Endoscopy 10.1007/s00464-021-08822-2

This article was updated to correct the units for “radiation exposure time” in Table 3.

